# Sensitive and frequent identification of high avidity neo-epitope specific CD8^**+**^ T cells in immunotherapy-naive ovarian cancer

**DOI:** 10.1038/s41467-018-03301-0

**Published:** 2018-03-15

**Authors:** Sara Bobisse, Raphael Genolet, Annalisa Roberti, Janos L. Tanyi, Julien Racle, Brian J. Stevenson, Christian Iseli, Alexandra Michel, Marie-Aude Le Bitoux, Philippe Guillaume, Julien Schmidt, Valentina Bianchi, Denarda Dangaj, Craig Fenwick, Laurent Derré, Ioannis Xenarios, Olivier Michielin, Pedro Romero, Dimitri S. Monos, Vincent Zoete, David Gfeller, Lana E. Kandalaft, George Coukos, Alexandre Harari

**Affiliations:** 1 Department of Oncology, Lausanne University Hospital, Ludwig Institute for Cancer Research, University of Lausanne, Lausanne, CH-1066 Switzerland; 2 Ovarian Cancer Research Center, Abramson Cancer Center, Perelman School of Medicine, University of Pennsylvania, Philadelphia, PA 19104 USA; 3 Swiss Institute of Bioinformatics, Lausanne, CH-1015 Switzerland; 4 Department of Medicine, Division of Immunology and Allergy, Lausanne University Hospital, Lausanne, CH-1066 Switzerland; 5 Urology Research Unit, Lausanne University Hospital, Lausanne, CH-1011 Switzerland; 6 Department of Pathology and Laboratory Medicine, Immunogenetics Laboratory, The Children’s Hospital of Philadelphia, Philadelphia, PA 19104 USA

## Abstract

Immunotherapy directed against private tumor neo-antigens derived from non-synonymous somatic mutations is a promising strategy of personalized cancer immunotherapy. However, feasibility in low mutational load tumor types remains unknown. Comprehensive and deep analysis of circulating and tumor-infiltrating lymphocytes (TILs) for neo-epitope specific CD8^+^ T cells has allowed prompt identification of oligoclonal and polyfunctional such cells from most immunotherapy-naive patients with advanced epithelial ovarian cancer studied. Neo-epitope recognition is discordant between circulating T cells and TILs, and is more likely to be found among TILs, which display higher functional avidity and unique TCRs with higher predicted affinity than their blood counterparts. Our results imply that identification of neo-epitope specific CD8^+^ T cells is achievable even in tumors with relatively low number of somatic mutations, and neo-epitope validation in TILs extends opportunities for mutanome-based personalized immunotherapies to such tumors.

## Abstract (web summary)

Epithelial ovarian cancer (EOC) has low mutational load. Here the authors analyze circulating and tumor-infiltrating lymphocytes (TILs) from 19 EOC patients and report frequent recovery of neo-antigen-reactive T cells from both compartments but with distinct TCR repertoires that have higher affinity in TILs.

## Introduction

Immunogenic tumors can benefit from different immunotherapeutic interventions. Among them, adoptive cell transfer (ACT) of autologous tumor-infiltrating lymphocytes (TILs) is effective in mediating tumor regression, especially in melanoma, where about half of patients can achieve an objective response and one-fourth of them can expect complete and durable tumor rejection^1,2^. Several clinical trials have explored the potential of antigen-specific T-cell therapy infusing autologous peripheral blood T cells engineered to express T-cell receptors (TCR) or chimeric antigen receptors specific for known shared tumor antigens^3–6^. Such advances have motivated widespread investigation of tumor rejection antigens across different tumor types and triggered the development of ACT approaches for the therapy of cancers other than melanoma. Recent technological advances have accelerated the identification of T-cell specificities against so-called tumor neo-antigens, resulting from non-synonymous somatic tumor mutations, and have shown their successful implication in immune-mediated rejection of melanoma, lung cancer, leukemia, and gastrointestinal cancers^7–12^. Several studies have also indicated that tumor neo-epitope recognition underlies clinical response in patients receiving immune checkpoint blockade therapy^9,11,13–15^ or ACT of autologous TILs^10,12,16–18^, although no direct correlation has been reported to date between the presence of documented neo-epitopes and patient survival in such studies. Tumors are very heterogeneous with regards to their mutational load^19^, and immune recognition of neo-antigens in tumors with relatively low mutational load is still considered unlikely^20^, thus limiting the potential application of mutanome-targeted immunotherapy.

Epithelial ovarian cancer (EOC) is a tumor with not only purportedly relatively low mutational load^19,21^, but also susceptible to immune recognition^22^. Spontaneous anti-tumor responses, both as tumor-specific lymphocytes and antibodies, were identified in about half of the patients with advanced EOC^23^, and cytotoxic T cells have been isolated from patients’ tumor, ascites, or peripheral blood^22^. Of note, the presence of CD8^+^ TILs has been linked to better prognosis in late-stage EOC patients^22,24^. Immunotherapy could thus be promising in EOC. However, vaccination strategies have to date mainly encompassed shared tumor-associated antigens and have been met with limited success^25^. With the exception of two interesting studies^21,26^, the landscape of spontaneous responses to tumor neo-epitope still has not been investigated thoroughly.

We investigated spontaneous recognition of tumor neo-epitopes in immunotherapy-naive, chemotherapy-pretreated patients with recurrent advanced EOC. We report the identification of neo-epitope specific CD8^+^ T cells in ~ 90% of patients evaluated. Culture conditions for TIL generation markedly influenced the sensitivity of detection and the frequency of neo-epitope specific T cells. Unexpectedly, neo-epitope recognition was largely discordant between circulating T lymphocytes and TILs, and the latter displayed markedly higher functional avidity. Of note, a deep molecular investigation of T cells sharing the same neo-epitope specificity, isolated from the two compartments, revealed distinct TCR repertoires, with higher affinity among TILs relative to blood T cells. Our data demonstrate that using sensitive methodologies, neo-epitope validation is achievable in low mutational load tumors, which extends opportunities for mutanome-based personalized immunotherapy for such patients.

## Results

### Identification of Neo-epitope specific PBL

We evaluated the neo-epitope landscape in 19 patients with recurrent advanced EOC who were immunotherapy-naive, but heavily pretreated with chemotherapy (Supplementary Table 1). Patients had no underlying inflammatory condition at enrollment and were not on steroids. We identified over 1300 non-synonymous somatic mutations in total by exome sequencing, with a median of 69 and a range of 10–129 mutations per patient. We used fetchGWI^27^ to maximize calls of non-synonymous somatic mutations and GATK to independently validate calls; fetchGWI calls overlapped up to 96% with GATK calls (see Methods). Using the NetMHC algorithm^28^, a total of 776 (9-mer or 10-mer) candidate neo-epitopes were predicted in silico to bind with high affinity to patients’ cognate HLA-I alleles, with a range of 1–133 neo-epitopes predicted per patient (Supplementary Table 1). As expected, the predicted neo-epitope load correlated with the overall tumor non-synonymous mutational load (*p* = 0.002, linear regression).

**Fig. 1:**
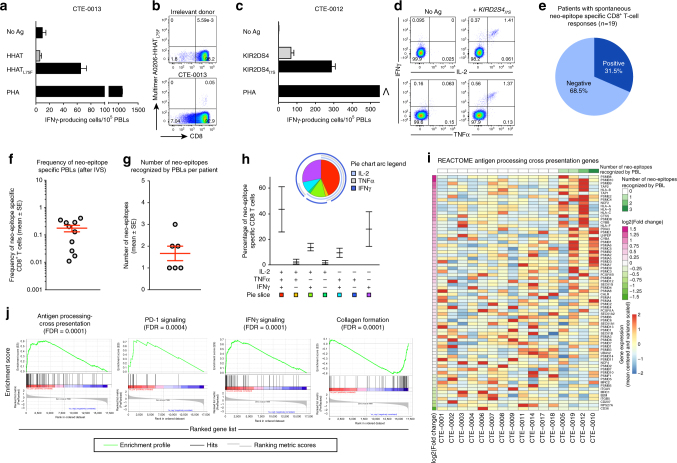
Identification of neo-epitope specific PBLs. **a**, **b** Representative example of peripheral blood CD8^+^ T lymphocytes (PBLs) response against the *HHAT*_*L75F*_ neo-epitope in patient CTE-0013. **a** T-cell reactivity measured by IFNγ ELISpot against the neo-epitope (mut) from *HHAT*_*L75F*_ gene but not against the wild-type (wt) peptide (PHA: phytohemagglutinin; No Ag: no peptide). Shown are the average of triplicate + SD. **b** Representative example of validation of neo-epitope specific CD8^+^ T cells using peptide-MHC multimers. **c** T-cell reactivity measured by IFNγ ELISpot against the neo-epitope from *KIR2DS4*_*I7S*_ gene but not against the wild-type peptide in patient CTE-0012 (PHA: phytohemagglutinin; No Ag: no peptide). Shown are the average of triplicate + SD. **d** Representative example of neo-epitope validation by polychromatic intracellular cytokine staining (ICS) in patient CTE-0012 without (No Ag) or after stimulation with a cognate neo-epitope (*KIR2DS4*_*I7S*_). All other PBL neo-epitopes are described in Supplementary Fig. 1 and Table 2. **e** Proportion of EOC patients with documented peripheral blood CD8^+^ T-cell (PBL) response against neo-epitopes. **f** Frequency and **g** number (mean ± SEM) of neo-epitope specific CD8^+^ PBLs per patient, detected after one round of in vitro stimulation (Supplementary Table 2). **h** Cumulative analysis showing the cytokine profiles of neo-epitope specific CD8^+^ PBLs. The SPICE analysis represents the functional composition of cytokine-producing neo-epitope specific CD8^+^ T cells (mean ± SEM). **i** Heatmap showing expression of all the genes from Reactome’s antigen processing and cross-presentation pathway^53, 54^. The number of neo-epitopes recognized by PBL of each patient is shown on top of the heatmap. Genes from this pathway are ordered based on the differential expression fold change between patients with or without PBL neo-epitope recognition. **j** Gene set enrichment analysis (GSEA^39^) curves for various Reactome pathways significantly different between patients with or without PBL neo-epitope recognition. Vertical bars indicate the ranked positions of each gene from the respective gene set. Statistical significance of these enrichments is reported by the FDR of the GSEA. Corresponding heatmaps for IFNγ signaling, PD-1 signaling and collagen formation are reported in Supplementary Fig. 2

To elucidate whether tumor neo-epitopes provided a basis for tumor immune recognition, we stimulated CD8^+^ peripheral blood lymphocytes (PBLs) with pools of predicted peptides for 12 days in vitro, followed by rechallenge with the same peptides and analysis by IFNγ ELISpot. T-cell responses against neo-epitopes were further validated by multimer staining and/or polychromatic intracellular cytokine staining (ICS). We identified PBLs recognizing HLA class-I neo-epitopes in one-third (i.e., 6/19) of the patients (Fig. 1a–e and Supplementary Fig. 1). Neo-epitope-specific CD8^+^ T cells did not recognize the counterpart wild-type peptides (Fig. 1a, c and Supplementary Fig. 1), had a broad range of frequencies (Fig. 1f) and were polyfunctional with most cells co-expressing IFNγ, TNFα, and IL-2 in response to the cognate neo-epitope (Fig. 1d, h). We validated a total of 10 non-overlapping neo-epitopes in 19 patients, with a range of 1–3 neo-epitopes validated per patient (Fig. 1g and Supplementary Table 2). Of note, the specificity and privacy of detection of neo-epitopes was validated in unrelated patients’ samples, supporting the notion that T-cell responses identified with our assay represented an expansion of pre-existing responses from antigen-experienced T cells and not priming of naive cells, although this cannot be formally excluded. In conclusion, T cells recognizing tumor neo-antigens can be specifically identified readily even in some patients with tumors harboring a relatively low mutational load such as ovarian cancer.

We searched for possible associations between spontaneous recognition of tumor neo-antigens and molecular signatures of tumors in 16/19 patients with tumor available for RNAseq analysis^29,30^. Tumors from patients in whom we could detect PBLs recognizing neo-epitopes displayed gene signatures significantly enriched for antigen processing and cross-presentation pathways as well as programmed cell death-1 and interferon-gamma (IFNγ) signaling, and negative enrichment for collagen formation (Fig. 1i, j and Supplementary Fig. 2 and Table 3). Thus, increased recognition of tumor neo-antigens by PBLs was associated with immune activation and an attenuated stroma signature at the tumor site. Interestingly, recognition of neo-antigens by PBLs was significantly higher in four patients harboring BRCA1/2 mutated tumors (*p* = 0.002, *χ*^2^, Supplementary Table 1), consistent with the higher mutational burden and predicted neo-antigen load found in these patients^31^, although the overall homologous recombination deficiency (HDR) status in this population was not determined.

### Identification of Neo-epitope specific TILs

**Fig. 2:**
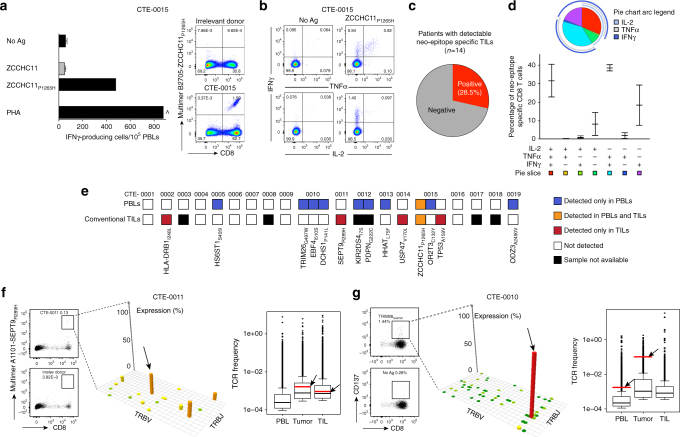
Identification of neo-epitope specific TILs. **a**–**c** Representative example of neo-epitope (*ZCCHC11*_*P1265H*_)-specific TILs in patient CTE-0015. **a** IFNγ ELISpot showing the average of triplicate + SD (PHA: phytohemagglutinin; No Ag: no peptide) and peptide-MHC multimer staining. All other neo-epitopes are described in Supplementary Fig. 3 and Table 2. **b** Intracellular cytokine staining showing the frequency of viable IFNγ, TNFα, and IL-2 cytokine-producing TILs. **c** Proportion of patients with neo-epitope reactive TILs. **d** Cumulative analysis (SPICE) showing the functional composition (cytokine profiles) of neo-epitope specific TILs (mean ± SD). **e** Repertoire of neo-epitopes in PBLs and TILs in 19 patients. Each colored square represents one neo-epitope for which T-cell reactivity was validated either exclusively in PBLs (blue), exclusively in TILs (red), in both compartments (orange) or in none of them (white). For 5 out of 19 patients no tumor samples were available for TIL generation (black squares). **f** Neo-epitope-specific TILs from patient CTE-0011 were FACS sorted with HLA-A1101-*SEPT9*_*R289H*_ multimer. Sorted cells (left) underwent TCR sequencing, as well as autologous bulk PBLs, bulk tumor and bulk TIL cultures. The Manhattan plot reports the V/J recombination of the T-cell receptor β (TCRβ) of *SEPT9*_*R289H*_-specific T cells: V and J segments are represented according to chromosomal location on the *x* and *y*-axis, respectively. On the right, the distribution of the TCR frequencies is shown for PBL, tumor and TILs. Dominant *SEPT9*_*R289H*_-specific TCRβ are identified by red bars (and arrows) in tumor and TILS, whereas no *SEPT9*_*R289H*_-specific TCRs was identified in PBLs. **g***TRIM26*_*G497W*_-specific circulating T cells from patient CTE-0010 were isolated based on CD137 upregulation and immediately processed for TCR-sequencing analysis (center). TCR repertoire analysis was performed in parallel on matched bulk PBLs, bulk tumor, and bulk TIL cultures (right). The most frequent shared neo-epitope specific TCR was ranked according to its relative frequency in PBL and tumor (red bars and arrows). The same TCR was undetectable in IL-2-expanded TIL cultures. Elements of box plots represent median (line), 25% and 75% confidence limit (box limits) and 10% and 90% confidence limit (whiskers)

We next asked what was the prevalence of neo-epitope recognition by TILs in the same patients. Tumor samples were available from 14 (out of 19) patients and we successfully expanded TILs from all patients using standard culture conditions (i.e., high-dose IL-2). TILs were interrogated with the same set of all predicted peptides for each patient as above, and T-cell responses were evaluated by IFNγ ELISpot and validated by ICS and/or multimer staining. Neo-epitope-specific TILs were identified in about one-quarter (i.e., 4/14) of patients (Fig. 2a–c and Supplementary Fig. 3). Like PBLs, a large proportion of neo-epitope specific TILs was also polyfunctional, i.e., able to produce IL-2, TNFα and IFNγ in response to the cognate neo-epitope (Fig. 2b, d).

In total, we detected PBLs or TILs recognizing neo-epitopes in two-thirds (9/14) of the patients, for a total of 14 distinct neo-epitopes (Fig. 2e and Supplementary Table 2). However, neo-epitope recognition was largely discordant between PBLs and TILs. Indeed, out of the 14 documented responses to neo-epitopes, only one was detected in both PBLs and TILs, whereas the 13 remaining neo-epitopes were exclusively recognized by either PBLs or TILs only (Fig. 2e).

We theorized that the discordance between detecting neo-epitope specific T cells in TILs but not in blood could be owing to a low frequency of neo-epitope specific T cells in blood. In a representative patient, who exhibited neo-epitope specific T cells exclusively in expanded TILs but not in PBLs, we sequenced the TCRβ of sorted CD8^+^ TILs specific to the confirmed neo-epitope (*SEPT9*_*R289H*_, Fig. 2f). As expected, neo-epitope specific CD8^+^ TILs were oligoclonal, and dominant TCRβ sequences were identified (Fig. 2f). These TCR sequences were detected in tumor tissue in addition to IL-2-expanded bulk TILs, but not among PBLs (Fig. 2f). Of note, >75,000 distinct clonotypes were identified among three millions reads obtained from 5 millions unfractionated total PBMCs and the limit of detection (LOD) of our assay was experimentally validated at 10 cells out of 1 million cells (i.e., 0.001%), suggesting that undetectable TCR were at a frequency below our LOD. To understand more the discordance of detecting neo-epitope specific T cells in blood but not in expanded TILs, we studied a representative patient by sequencing TCRβ of sorted CD8^+^ PBLs specific to the confirmed neo-epitope (*TRIM26*_*G497W*_, Fig. 2g). Interestingly, the dominant TCRβ specific to *TRIM26*_*G497W*_ neo-epitope that we detected in sorted PBLs, was also detected in whole tumor RNA, but was undetectable among more than 1900 distinct clonotypes identified within 1 million reads obtained from five millions IL-2-expanded TILs (Fig. 2g). Of note, the *TRIM26*_*G497W*_-specific TCR was among the most frequent TCRs in tumor tissue, indicating marked clonal expansion in tumor (Fig. 2g). Thus, neo-epitope specific T cells detected in blood may infiltrate and expand in tumors, but may fail to expand under standard TIL cultures, or at least may not reach a frequency allowing their detection.

### Cultures enrichment for neo-epitope specific TILs

**Fig. 3:**
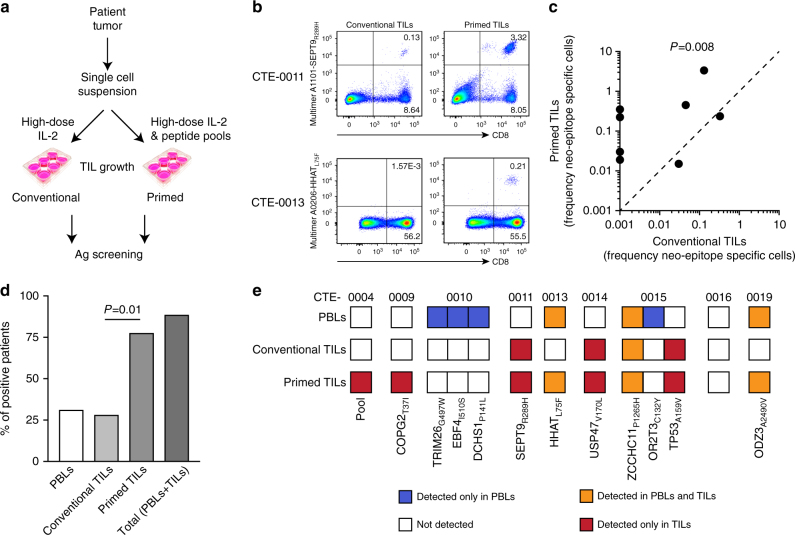
TIL cultures can be enriched for neo-epitope specific T cells. **a** Schematic representation of the alternative procedures for TIL expansion. Single cell tumor suspensions were plated in the presence of high-dose IL-2 alone (conventional TILs) or alternatively, in the presence of high-dose IL-2 supplemented with pools of predicted mutated peptides (primed TILs). **b** Representative examples of conventional and primed TILs, interrogated for the presence of neo-epitope specific TILs by multimer staining. **c** Cumulative analysis of the frequencies of neo-epitope specific CD8^+^ T lymphocytes detected in conventional (*x-*axis) and primed (*y*-axis) TIL cultures. **d** Proportions of patients with documented neo-epitope specific T-cell responses in the different compartments and within the distinct TIL culture conditions. **e** Landscape of neo-epitope specific T-cell responses in circulating, conventional TILs and primed TILs in nine patients for which primed TILs were available. T-cell responses against mutated epitopes identified exclusively in PBLs (blue), in TILs (red), in both the compartments (orange) or in none of them (white) are indicated

Although the conventional TIL expansion methods have served well patients with melanoma, which typically express numerous neo-epitopes, the above findings suggest that for tumors with low average mutational load, optimization of TIL cultures will be required to maximize the recovery of neo-epitope specific T-cell clones. To this end, we sought to optimize the ovarian TIL expansion to favor expansion of neo-epitope specific clones. We expanded TILs from nine patients with available samples either in high-dose IL-2 alone (conventional TIL generation) or also in the presence of pools of synthetic 9- and 10-mer peptides of all predicted class-I neo-antigens (neo-epitope primed TIL condition, Fig. 3a). The different culture conditions led to similar expansion rates of bulk T cells and CD4^+^/CD8^+^ T-cell ratios. However, compared with TILs cultured under conventional conditions, neo-epitope primed TILs were enriched in neo-epitope specific T cells, including markedly higher frequencies of CD8^+^ T-cell clones recognizing either the same neo-epitope (Fig. 3b, top panels) or new neo-epitopes that were undetectable under the conventional expansion protocol (Fig. 3b, bottom panels and Supplementary Fig. 3).

Taken together, the primed TIL cultures were significantly enriched in neo-epitope specific CD8^+^ T cells as compared with conventional TILs generated from the same patients (*χ*^2^, Fig. 3c). Furthermore, as a result of the primed culture condition, we were able to identify neo-epitope specific TILs in a significantly higher number of patients, and combining PBLs and TILs expanded with the improved methodology, we detected T cells against neo-epitopes in most patients (i.e., 8/9, Fig. 3d). The specificity of neo-epitope detection was validated in cross-matching experiments. These data show that with optimized culture methods, T cells recognizing tumor neo-epitopes can be specifically identified in most patients with ovarian cancer despite the relatively low number of somatic mutations. Of note, even with the expanded ability to identify T-cell responses against neo-epitopes, neo-epitope recognition remained largely discordant between PBLs and TILs. Indeed, only a quarter of neo-epitopes (i.e., 3/12) was consistently recognized by both PBLs and TILs (Fig. 3e).

### Higher avidity of neo-epitope specific TIL than PBL clones

**Fig. 4:**
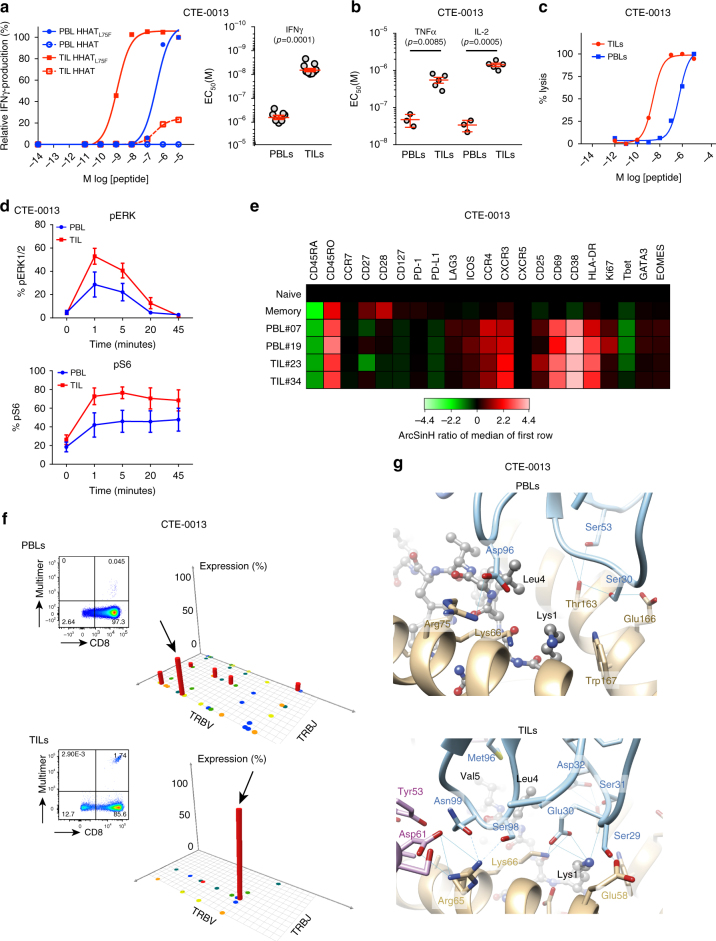
Functional analyses of neo-epitope specific PBLs and TILs. **a** Higher functional avidity of *HHAT*_*L75F*_-specific TIL and PBL clones. Shown are the relative frequencies of IFNγ-producing CD8^+^ T cells (mean ± SD). **b** Higher functional avidity (mean ± SEM) of *HHAT*_*L75F*_-specific TILs compared with PBL clones based on TNFα and IL-2 release (Supplementary Fig. 5 shows raw data). **c** Higher cytolytic capacity of *HHAT*_*L75F*_-specific TILs compared with PBLs. Data show the percentage of lysis of T2 cells pulsed with different concentrations of the cognate neo-epitope. **d** Mass cytometry analysis showing the relative phosphorylation of ERK1/2 and S6 proteins involved in TCR-signaling pathways following CD3 stimulation of PBL and TIL clones. Graphs represent mean ± SEM. Supplementary Fig. 6 shows raw data. **e** Mass cytometry analysis showing the relative expression of different markers on neo-epitope specific PBLs and TILs. Naive and memory bulk peripheral blood CD8^+^ T cells are shown for comparison. **f** Analysis of the repertoire of the T-cell receptor β (TCRβ) V-J segment recombination of *HHAT*_*L75F*_-specific CD8^+^ T cells isolated from either PBLs (top) or TILs (bottom) after FACS sorting using multimers. V and J segments are represented according to chromosomal location on the *x* and *y*-axis, respectively. Arrows identify dominant TCRβ sequences that were associated to TCRα sequences shown in Supplementary Fig. 8. **g** Calculated TCR/pMHC complexes for PBL and TIL-related TCRs: *HHAT*_*L75F*_-PBL-hTRAV05 + hTRAJ34/hTRBV11-2 + hTRBJ02-7 on the top, and *HHAT*_*L75F*_-TIL-hTRAV38-2 + hTRAJ33/hTRBV12-3 + hTRBJ02-3 at the bottom. The peptide is shown in ball and stick, colored according to the atom types. MHC ribbon is colored in brown, with residues displayed in thick lines and colored according to the atom types, with carbon colored in brown. TCRα ribbon is colored in light blue, with residues displayed in thick lines and colored according to the atom types, with carbon colored in light blue. TCRβ ribbon is colored in pink, with residues displayed in thick lines and colored according to the atom types, with carbon colored in pink. Hydrogen bonds and ionic interactions are shown as thin blue lines. Dotted blue lines indicate hydrogen bonds accessible through thermal fluctuations. Residues are labeled in brown, black, blue, and pink, for MHC, peptide, TCRα, and TCRβ, respectively

We investigated further the discordance between neo-epitope specific PBLs and TILs by first focusing on two neo-epitopes that were recognized by both PBLs and TILs, i.e., *HHAT*_*L75F*_ from CTE-0013 and *ZCCHC11*_*P1265H*_ from CTE-0015. We purified neo-epitope reactive T cells from PBLs and TILs using multimers and expanded several clones of each. We measured functional avidity of 3–6 clones for each by measuring their sensitivity to cognate neo-epitope, i.e., the peptide concentration sufficient to evoke IFNγ response in 50% of cells. All TIL clones had significantly higher functional avidity compared with PBL clones recognizing the same neo-epitope in each case (*t*-test, Fig. 4a and Supplementary Fig. 4), further confirmed by significantly higher TNFα and IL-2 secretion (*t*-test, Fig. 4b and Supplementary Fig. 5) and by higher cytolytic activity against T2 cells loaded with cognate peptide (Fig. 4c). Multidimensional mass cytometry analyses were performed on several clones to further characterize their phenotype. Consistent with the above, TILs exhibited significantly higher phosphorylation of ERK1/2 and S6 (and similar phosphorylation of ZAP70, SLP76, and CREB) than PBLs in response to peptide recognition (*t*-test, Fig. 4d and Supplementary Fig. 6). Further characterization of these clones showed a similar phenotype with regard to markers of differentiation, activation, exhaustion, or tropism (Fig. 4e), and expressed a similar pattern of cytokines following CD3/CD28 costimulation (Supplementary Fig. 7).

To further understand the differences in avidity between TIL and PBL clones, we sequenced the TCRβ of all bulk-specific cells sorted from blood or TILs against the neo-epitope *HHAT*_*L75F*_ in CTE-0013 and *ZCCHC11*_*P1265H*_ in CTE-0015. We found oligoclonal repertoires in both PBLs and TILs, but no common TCRβ sequences between TILs and PBLs directed against the same epitope in both cases analyzed (Fig. 4f and Supplementary Fig. 4). We then asked whether the different avidity between PBL and TIL clones could be potentially attributed to different TCR affinities. Having the cognate TCRα and β chain sequences for PBL and TIL clones (two α and one β chain were identified for each, see Supplementary Fig. 8 and Table 4) as well as the cognate peptide and HLA sequence available, we used homology modeling to predict molecular interactions between the dominant TCRs in PBLs or TILs with the pMHC complex formed by the *HHAT*_*L75F*_ neo-epitope and HLA-A*0206. These molecular interactions are expected to correlate qualitatively with the affinities of these TCRs for the pMHC^32^. The predicted molecular interactions between the *HHAT*_*L75F*_ peptide and HLA-A*0206 were similar for all four TCR-pMHC complexes analyzed (Supplementary Fig. 9 and Table 5). Of note, peptide residues Lys1, Leu4, Val5, and Phe8 were predicted to be exposed to the TCR surface (Fig. 4g and Supplementary Fig. 9). Predicted interactions between each of the four TCRs studied and the peptide/HLA-A*0206 complex are listed in Supplementary Table 6. According to these predictions, the CDR3α and CDR3β loops of the TIL TCRs lead to more numerous favorable molecular interactions with the peptide residues (i.e., eight and six) than the PBL TCRs (i.e., one and four). In addition, the CDR1α, CDR2α, CDR1β, and CDR2β of the PBL TCRs make a total of five and eight interactions with the peptide, compared with 11 between the TILs TCRs and the peptide. Finally, the PBL TCRs were predicted to make a total of 15 and 18 favorable interactions with the MHC, compared with 20 and 23 interactions for the TIL TCRs (Supplementary Table 6). In summary, TIL TCRs were predicted to make qualitatively more numerous favorable interactions with the pMHC than PBL TCRs, which is expected to correlate with a higher affinity of the TIL TCRs for this pMHC. Taken together the above data indicate that TILs exhibit markedly higher functional avidity against neo-epitopes, and this can be attributed to the accumulation of distinct clones with predicted higher affinity TCRs although we cannot formally exclude that other parameters such as peptide affinity and T-cell exhaustion may also contribute to this effect.

**Fig. 5:**
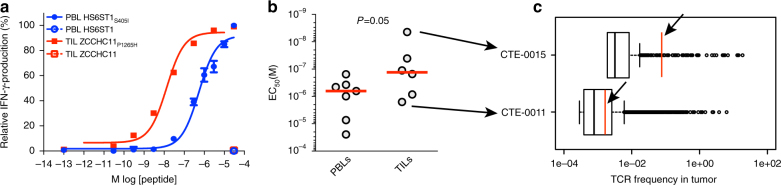
Functional analyses of neo-epitope specific PBLs and TILs. **a** Representative analyses of the functional avidity of neo-epitope specific PBLs and TILs as assessed by IFNγ ELISpot. *HS6ST1*_*S405I*_-specific PBLs from patient CTE-0005 and *ZCCHC11*_*P1265H*_-specific TILs from patient CTE-0015 were stimulated with serial dilutions of cognate neo-epitopes or native (wt) peptides. Data show mean ± SEM of technical replicates. **b** Cumulative analysis (individual values and median) of the functional avidity of neo-epitope specific CD8^+^ PBLs and TILs (raw data are shown in Supplementary Fig. 10). **c** Analysis of *ODZ3*_*A2490V*_-specific (top, high avidity) and *SEPT9*_*R289H*_-specific (bottom, low-avidity) TCRs (red bars and arrows) among total TCRs from the autologous tumor samples of patients CTE-0015 and CTE-0011, respectively. Elements of box plots represent median (line), 25% and 75% confidence limit (box limits) and 10% and 90% confidence limit (whiskers)

Finally, we analyzed the functional avidity of PBL and TIL clones specific to the remaining neo-epitopes. We found that TIL clones displayed on average a significantly higher functional avidity than PBLs (*t*-test, Fig. 5a, b and Supplementary Fig. 10), with the median avidity of TILs being almost one order of magnitude higher than that of PBLs (Fig. 5b). Finally, we asked whether the ex vivo functional avidity of TILs correlated with their frequency in tumors. We therefore purified and sequenced the TCRβ of the highest avidity TIL clone from CTE-0015 recognizing the *ZCCHC11*_*P1265H*_ neo-epitope and the lowest avidity TIL clone against *SEPT9*_*R289H*_ from CTE-0011 and searched for the specific TCR sequences within the tumors of origin (Fig. 5c). As shown already (Fig. 2f, g), neo-epitope specific CD8 T cells were oligoclonal although dominant TCRβ sequences could be identified. Of interest, we found that the high-avidity *ZCCHC11*_*P1265H*_-specific TIL dominant clone was markedly more expanded in tumor (1% of all tumor TCRs) than the low-avidity *SEPT9*_*R289H*_-specific TIL clone (0.001% of all tumor TCRs, Fig. 5c). Thus, neo-epitope specific TILs exhibit in general higher avidity for cognate neo-antigens relative to PBLs and can be found in the tumor at relatively high frequency.

## Discussion

We analyzed neo-epitope recognition in a cohort of immunotherapy-naive patients with epithelial ovarian cancer. Although this is a tumor type with relatively low mutational load in agreement with prior reports^19,21^, we identified T cells recognizing neo-epitopes in the majority of patients, consistent with prior evidence that ovarian cancers are susceptible to immune recognition^22^. Through blood analysis we identified circulating T-cell precursors recognizing neo-epitopes in one-third of the patients, whereas the identification of these cells was more frequent using TILs. Indeed, using an optimized methodology for TIL expansion that enriched for neo-epitope specific T cells, these could be identified in virtually all patients with available blood and tumor to isolate T cells. Of note, our findings have likely underestimated the prevalence of neo-epitope specific T cells in blood and tumors as our discovery approach was restricted to CD8^+^ T cells, therefore missing a potentially significant CD4^+^ T-cell response^7,12^. Also, we were unable to generate autologous tumor cell lines, so we could not confirm that the neo-antigen-reactive T cells recognize autologous tumor.

It is interesting that circulating neo-epitope specific T cells were identified only in a proportion of patients, specifically those whose tumors exhibited molecular evidence of immune activation and BRCA1/2 deficiency. This is in agreement with prior evidence that tumor-specific T-cell precursors can be identified only in a subset of patients with ovarian cancer^23^, and suggests that the frequency of these precursors is higher in patients in whom proper antigen presentation and a rejection response occur in tumors. On the other hand, our data suggest that antigen recognition may occur and neo-epitope specific T cells can accumulate in all tumors, even in those where a bona fide rejection response cannot be detected. Such cells could in the future be used for developing ACT.

We provide novel evidence that the repertoire of neo-epitope recognizing T cells can be largely discordant between blood and TILs. Importantly, neo-epitope specific TILs exhibited on average higher functional avidity than their blood counterparts. This was confirmed when we compared T cells from blood and tumor recognizing the same neo-epitopes: TILs exhibited much higher functional avidity and cytolytic activity than their blood counterparts, which was attributed to the presence of different clones in the two compartments with different TCR affinities. These findings have important implication for understanding tumor immunobiology but also for developing adoptive T-cell therapy. Importantly, neo-epitope specific clones exhibited suitable signaling in response to cognate antigen, and following CD3/CD28 costimulation their phenotype was quite similar to peripheral blood T cells, rendering them therefore a preferred source for isolating neo-epitope specific clones for ACT, which our data show can be feasible even in tumors with relatively low number of somatic mutations, extending opportunities for mutanome-based personalized immunotherapies.

## Methods

### Human subjects

Patients with recurrent ovarian, fallopian tube, or primary peritoneal grade 2 and 3 cancer who had received several lines of chemotherapy (Supplementary Table 1) were enrolled under protocols approved by the respective institutional regulatory committees at the University of Pennsylvania (Penn), USA, and Lausanne university hospital (CHUV), Switzerland. In particular, none of the subjects had any underlying infection or inflammatory condition at the time of study enrollment. Informed consent was obtained from all patients. All immune analyses were conducted at the Lausanne Branch of the Ludwig Institute for Cancer Research (LLB).

### Identification of non-synonymous tumor mutations

Genomic DNA from cryopreserved tumor tissue and matched PBMC was isolated using DNeasy kit (Qiagen) and subjected to whole-exome capture and paired-end sequencing using the HiSeq2500 Illumina platform. Data analysis was performed at the Vital-IT Systems Biology Division, Swiss Institute of Bioinformatics (SIB), Lausanne. Somatic variants were called from the exome reads and the reference human genome hg19 by using a software pipeline composed of a genome mapping tool, fetchGWI^27^, followed by a detailed sequence alignment tool, align0. Non-deterministic predictors of any kind were avoided and the route of minimizing false negative was prioritized and a cross-comparison with GATK as consensual variant detection/prediction method reached over 96% agreement. Variations present in the tumor samples and absent from the corresponding blood samples were assumed to be somatic.

### Neo-epitope prediction

Binding predictions to class-I HLA alleles for all candidate peptides incorporating somatic non-synonymous mutations were performed using the netMHC algorithm v3.4^28^. Candidate neo-epitope peptides (i.e., mutant 9-mer and 10-mer peptide sequences containing the somatically altered residue at each possible position) with a predicted binding affinity of ≤ 500 nM, and their wild-type native predicted peptides were synthesized (at > 90% HPLC purity) at the Protein and Peptide Chemistry Facility (PPCF), University of Lausanne.

### Identification of circulating neo-epitope specific T cells

CD8^+^ T cells (10^6^ mL^−1^) isolated (Dynabeads, Invitrogen) from cryopreserved PBMC were co-incubated with autologous irradiated CD8^+^- and CD4^+^-depleted PBMCs and peptides (1 µg mL^−1^, single peptide, or pools of ≤ 50 peptides) in RPMI supplemented with 8% human serum and IL-2 (20 IU mL^−1^ for 48 h and then 100 IU mL^−1^). IFNγ Enzyme-Linked ImmunoSpot (ELISpot), peptide-MHC multimer complexes staining and ICS assays were performed at day 12. T-cell reactivity for every neo-epitopes was validated by ≥ 2 independent experiments.

ELISpot assays were performed using pre-coated 96-well ELISpot plates (Mabtech) and counted with Bioreader-6000-E (BioSys)^33^. We considered as positive conditions those with an average number of spots higher than the counts of the negative control (No Ag) plus 3 times the standard deviation of the negative. For ICS, T cells were plated with CD4^+^ blasts in a 1:1 ratio and brefeldinA (BD biosciences, USA). After 16–18 h, cells were harvested and stained with anti-CD3, anti-CD8, anti-CD4, anti-IL-2, anti-TNFα, anti-IFNγ (BD biosciences) and with viability dye (Life technologies), acquired on a four-lasers Fortessa (BD biosciences), and analyzed with FlowJo X (TreeStar) and SPICE 4.2.3^34^. The number of lymphocyte-gated events ranged between 10^5^ and 10^6^.

### TILs expansion and interrogation

TILs were generated from tumor enzymatic digestion by plating total dissociated tumor in p24-well plates at a density of 1 × 10^6 ^cells per well in RMPI supplemented with 8% human serum and hrIL-2 (6000 IU mL^−1^) without (conventional) or with (primed) 1 µg mL^−1^ of predicted peptides (in pools of ≤ 50 peptides). After 2–4 weeks, TILs were collected and a fraction of the cultures underwent a rapid expansion (REP) for 14 days. T-cell reactivity against predicted neo-antigens was tested by IFNγ ELISpot on pre-REP TILs when available and post-REP TILs as described above. Positivity was confirmed in ≥ 2 independent experiments.

### Isolation and expansion of neo-epitope specific T cells

Circulating and tumor-infiltrating neo-epitope specific CD8^+^ T cells were FACS sorted using in-house reversible multimers (NTAmers^35^), and were either used for TCR sequencing (see below) or expanded or cloned by limiting dilution. To this end, cells were plated in p96-well plates and stimulated with irradiated feeder cells (PBMC from two donors) in RPMI supplemented with 8% human serum, phytohemagglutinin (1 μg mL^−1^) and IL-2 (150 U mL^−1^). At the end of the REP, multimer-positive cells were > 95% pure.

### Functional avidity

Functional avidity of neo-epitope specific CD8^+^ T-cell responses was assessed by performing limiting peptide dilutions (ranging from 30 µg mL^−1^ to 0.3 pg mL^−1^) in in vitro IFNγ ELISpot assays or Meso Scale Discovery (MSD) platform (see below). The peptide concentration required to achieve a half-maximal cytokine response (EC_50_) was determined^36^.

### Mass cytometry (CyTOF) analyses

Cryopreserved PBL and TIL clones were thawed, rested, and then stained using metal-conjugated antibodies according to the CyTOF manufacturer’s instructions (Fluidigm, San Francisco, CA). Samples were pooled and stained using a cocktail of antibodies for cell surface markers, washed with cell staining media (CSM) and phosphate-buffered saline (PBS), fixed with 2.4% formaldehyde, washed with CSM-S, and stained for intracellular targets. All antibodies are described in Supplementary Methods. After intracellular staining, cells were resuspended in DNA-intercalation solution (PBS, 1 μM Ir-Intercalator, 1% formaldehyde, 0.3% saponin) and stored at 4 °C until analysis. For intracellular cytokine stainings, cells were mixed with beads coated with αCD3 and αCD28 antibodies and incubated overnight in presence of brefeldinA (BD). Cells were then washed, and stained as discussed above. TCR signaling studies were performed by pre-incubating PBL or TIL clones with 1 μg mL^−1^ αCD3 for 5 min at 37 °C followed by the addition of avidin to initiate T-cell activation. Aliquots of cells were removed at the indicated times and the cellular phosphorylation states were preserved by fixing the cells with 2% formaldehyde in PBS at 4 °C. Cells were permeabilized with ice cold 90% methanol and individual timepoints were stained with isothiocyanobenzyl-ethylenediaminetetraacetic acid loaded with one of five palladium isotopes (104, 105, 106, 108, 110 u obtained from Trace Sciences, Canada). Palladium-labeled cells were washed with CSM and all samples were pooled prior to cell staining with a cocktail of antibodies for cell surface and intracellular phosphoproteins, described in Supplementary Methods. DNA staining and cell preparation were performed as discussed above. For CyTOF analysis, cells were washed three times with MilliQ water and resuspended at 0.5 × 10E6 cells mL^−1^ in 0.1% EQ Four Element Calibration Beads solution (Fluidigm). Samples were acquired on an upgraded CyTOF using a syringe pump at 45 μL per min. FCS files were concatenated and normalized using the cytobank concatenation tool and matlab normalizer, respectively. Data were processed and analyzed with cytobank and R using the OpenCyto and cytofkit packages.

### Cytokines measurement

Cytokines measurement of culture supernatants was performed using the human T_H_1/T_H_2 10-Plex tissue culture kit from MSD platform. Supernatants of 96-well culture plates of stimulated and unstimulated lymphocytes were analyzed using standard multiplex plates as per the manufacturer’s instructions.

### In vitro killing assay

Peptide-loaded, ^51^Cr labeled target T2 cells were co-incubated for 4 h at 37 °C with T cells (neo-epitope specific PBLs and TILs sorted and expanded as above) at a ratio of 1:10. At the end of the co-culture, supernatant was collected and analyzed for radio-reactivity using Topcount Instrument (Perkin Elmer). The percentage of specific lysis was calculated as: 100 × [(experimental−spontaneous release)/(total−spontaneous release)].

### TCRα and TCRβ sequencing

Total RNA was isolated using the RNeasy Micro Kit (Qiagen) and mRNA was then amplified using the MessageAmp II aRNA Amplification Kit (Ambion) with the following modifications: 500 ng of total RNA was used as starting material. The in vitro transcription was performed at 37 °C for 16 h. First-strand complementary DNA was synthesized using the Superscript III (Thermofisher) and a collection of TRAV/TRBV-specific primers. TCRs were then amplified by PCR (20 cycles with the Phusion from NEB) with a single primer pair binding to the constant region and the adapter linked to the TRAV/TRBV primers added during the reverse transcription. A second round of PCR (25 cycles with the Phusion from NEB) was performed to add the Illumina adapters containing the different indexes. The TCR products were purified with AMPure XP beads (Beckman Coulter), quantified and loaded on the MiniSeq instrument (Illumina) for deep sequencing of the TCRα/TCRβ chain. The TCR sequences were further processed using ad hoc Perl scripts to: (i) pool all TCR sequences coding for the same protein sequence; (ii) filter out all out-frame sequences; (iii) determine the abundance of each distinct TCR sequence. TCR with a single read were not considered for the analysis.

### Gene expression analysis

High quality RNA (1 μg) was subjected to paired-end sequencing using the HiSeq2500 Illumina platform. Complementary DNA libraries were constructed using mRNA-Seq Sample Prep Kit based on the Illumina guide. Library size distribution was validated using Agilent Technologies 2100 Bioanalyzer and quantified by quantitative PCR (KAPA Library Quant Kits, KAPA biosystem). Four normalized sample libraries were pooled together and loaded to a single lane of an Illumina flow cell. Data analysis was performed at the Vital-IT Systems Biology Division, SIB, Lausanne, and at the Lausanne Branch of the Ludwig Institute for Cancer Research. Differential gene expression between groups was performed with DESeq2^37^ in R. The genes were ranked based on the fold change between patients with or without PBL neo-epitope recognition and this ranked gene list was inputted into GSEA^38^ to perform gene set enrichment analysis. For this analysis, the enrichment for all the “Canonical pathways” gene sets (version 5.1) from the “Molecular Signature Database”^39^ was tested (some results are highlighted in Fig. 1i,j and Supplementary Fig. 2; all results with an FDR below 0.05 are reported in Supplementary Table 3).

### TCR-pMHC structure modeling

The protocol used to model the TCR-p-MHC complexes was adapted from our TCRep 3D approach^32^. Starting from V and J segment identifiers and from the CDR3 sequences, the full sequence of the constant and variable domains of TCRα and TCRβ were reconstituted based on IMGT/GENE-DB reference sequences^40^. Homology models of the TCR-p-MHC complexes were obtained using the Modeller^41,42^ program, version 9. Template experimental structures were taken from the Protein Data Bank^43^, and selected based on the sequence similarity to the different components of the complexes, i.e., peptide, MHC, β-microglobulin, TCRα, and TCRβ (Supplementary Table 4). Sequence alignments between the target and template proteins were obtained using the MUSCLE^44,45^ program. A total of 500 models were produced for each TCR-p-MHC complex, and ranked according to the Modeller Objective Function. The best ranked model was selected for CDR loop refinement. The later was performed by creating 4 × 500 alternative loop conformations using the “loop modeling” module of Modeller. During this refinement, loops were treated by pairs, as follows: TCRα CDR1 and CDR3 were optimized simultaneously by creating 500 loop conformations (whereas other CDR loops were held fixed), followed by TCRα CDR1 and CDR2, TCRβ CDR1 and CDR3 and finally TCRβ CDR1 and CDR2, in this order. After each of these four loop refinement steps, all models were ranked according to the Molecular Mechanics—Generalized Born Surface Area (MM-GBSA) score we used previously to perform TCR engineering^46–48^. The total energy of the system was calculated using the CHARMM27^49^ force field, and the CHARMM v39 molecular mechanics package^50^. The electrostatic solvation free energy was calculated using the GB-MV2^51^ implicit solvent model, with a dielectric of 1 and 80 for the protein and solvent, respectively, and no cutoff on the non-bonded terms. The non-polar solvation energy was estimated by weighting the solvent accessible surface area calculated analytically with CHARMM (with a probe radius of 1.4 Å) by a 0.0072 kcal/mol/Å^2^ surface tension. After each step of loop refinement, the model with the most favorable MM-GBSA energy was selected for the next step. Molecular graphics and analyses were performed with the UCSF Chimera package^52^.

### Statistical analyses

Differences between averages of variables were compared using two-tail *t*-test for variables with normal distribution or by using Mann–Whitney non-parametric test for non-normal variables. Analyses of contingency were performed by Fisher’s exact test using Graphpad PRISM 7.0.

### Data availability

The exome and RNA sequencing data have been deposited in the the European Genome-phenome Archive (EGA) database under the accession code EGAS00001002803. The authors declare that all the other data supporting the findings of this study are available within the article and its supplementary information files and from the corresponding authors upon reasonable request.

## Electronic supplementary material

Supplementary Information(DOCX 88402 kb)
