# Supplementary material for: Sensitive and frequent identification of high avidity neo-epitope specific CD8+ T cells in immunotherapy-naive ovarian cancer
Source: Nat Commun. 2018 Mar 15;9:1092. doi: 10.1038/s41467-018-03301-0 (PMC5854609; doi:10.1038/s41467-018-03301-0)
Supplement: Supplementary file 1 — Supplementary Information(DOCX 88402 kb) [file 41467_2018_3301_MOESM1_ESM.docx]

**SUPPLEMENTARY INFORMATION**

**Sensitive and Frequent Identification of High Avidity Neo-epitope Specific CD8^+^ T-cells in Immunotherapy-naïve Ovarian Cancer**

**Bobisse et al.**

**Supplementary Figure 1**

**
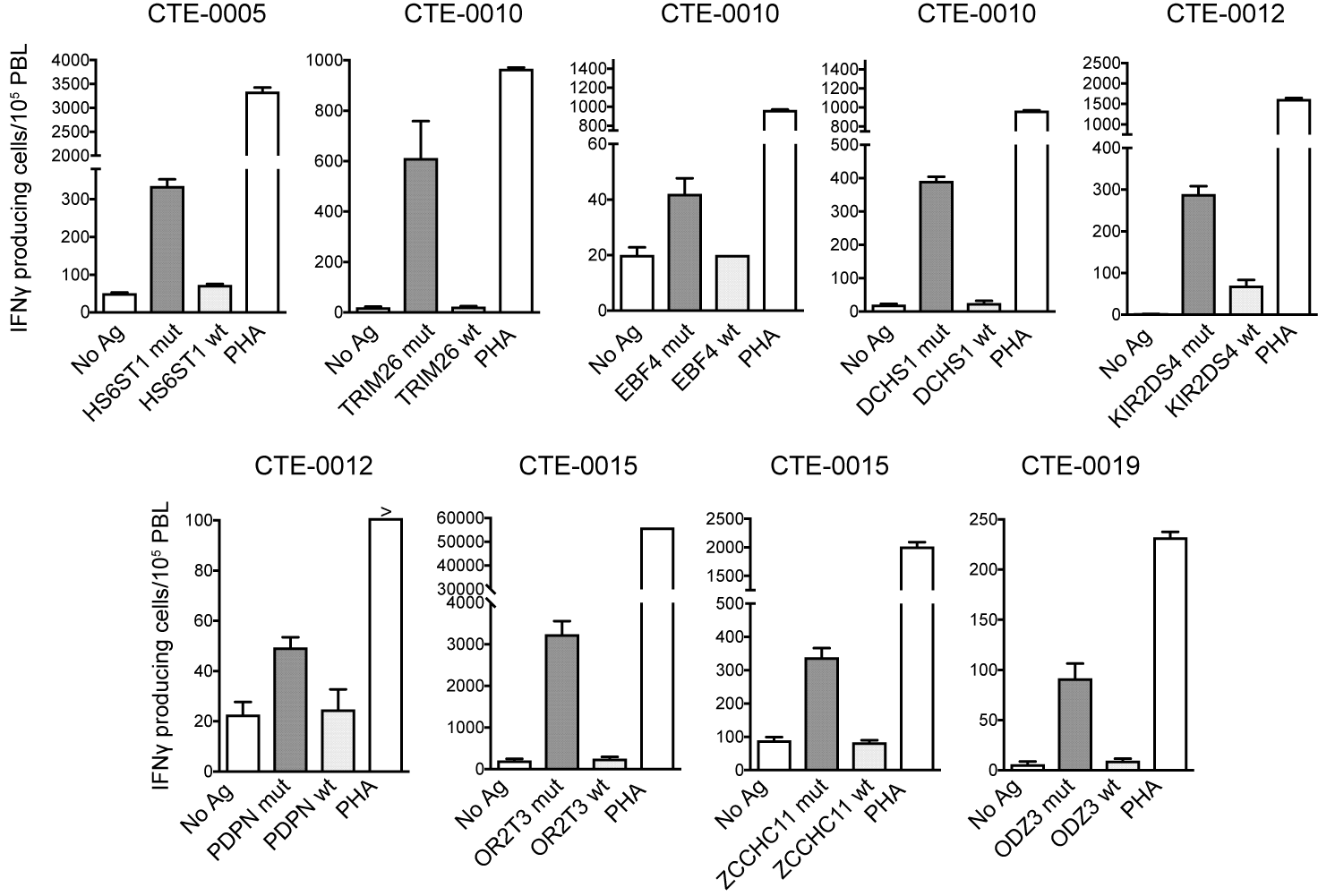
**

**Supplementary Figure 1. Validations of all peripheral blood CD8^+^ T-cell responses against neo-epitopes and native (wild-type) peptides**

Shown are the numbers of IFNγ-producing CD8^+^ T cells determined by ELISpot (average of triplicates + SD), upon ≥ one round of *in vitro* stimulation and tested against individual positive peptides. Neo-epitopes are described in Supplementary Table 2.

**Supplementary Figure 2**

**
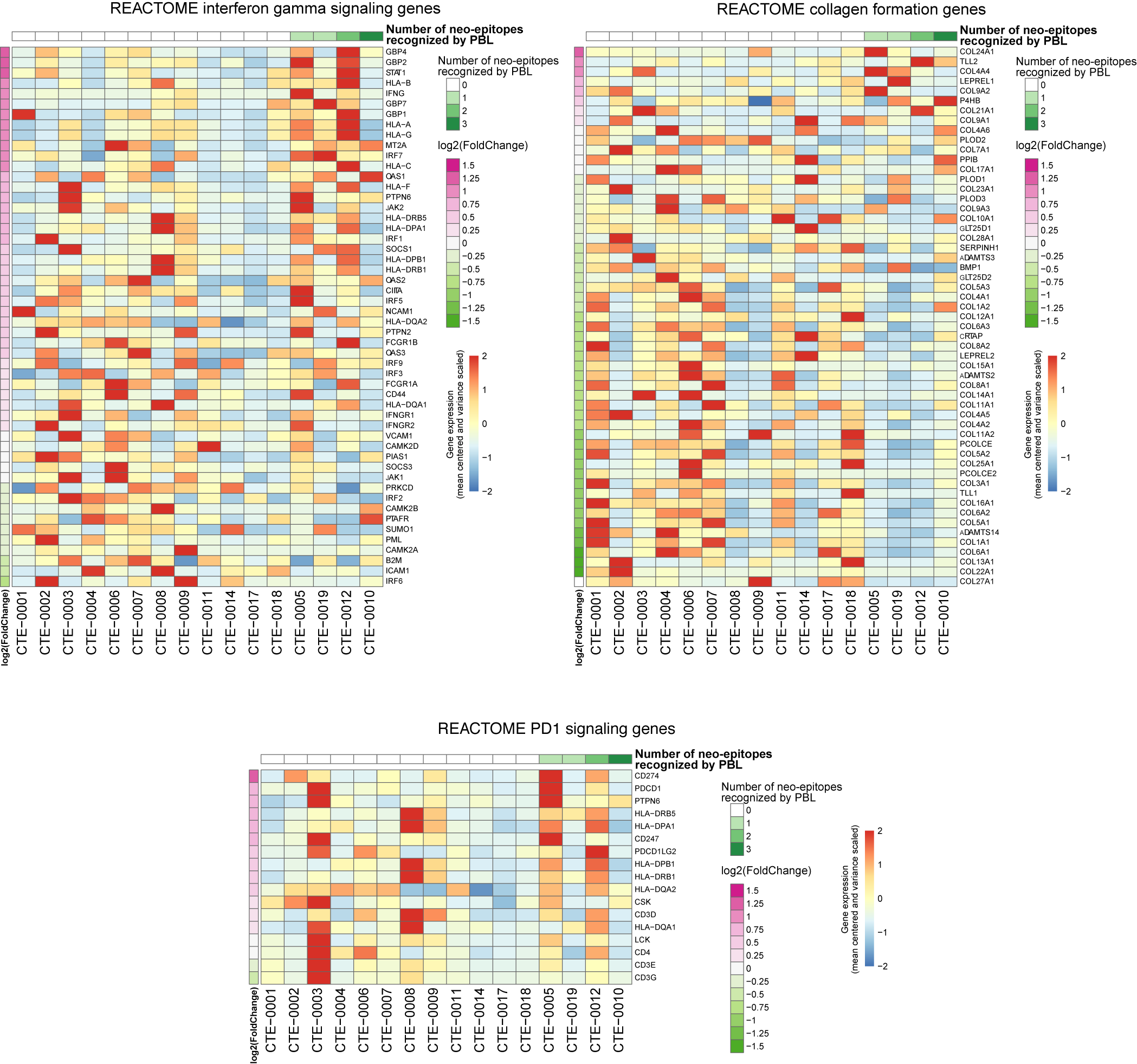
**

**Supplementary Figure 2. Heatmaps showing expression of genes related to PD-1 signaling, IFNγ signaling and collagen formation pathways**

The number of neo-epitopes recognized by PBLs of each patient is shown on top of the heatmaps. Genes from each pathway are ordered based on the differential expression fold change between patients with or without PBL neo-epitope recognition. Corresponding gene set enrichment analysis curves for these pathways are shown in Fig. 1i.

**Supplementary Figure 3**

**
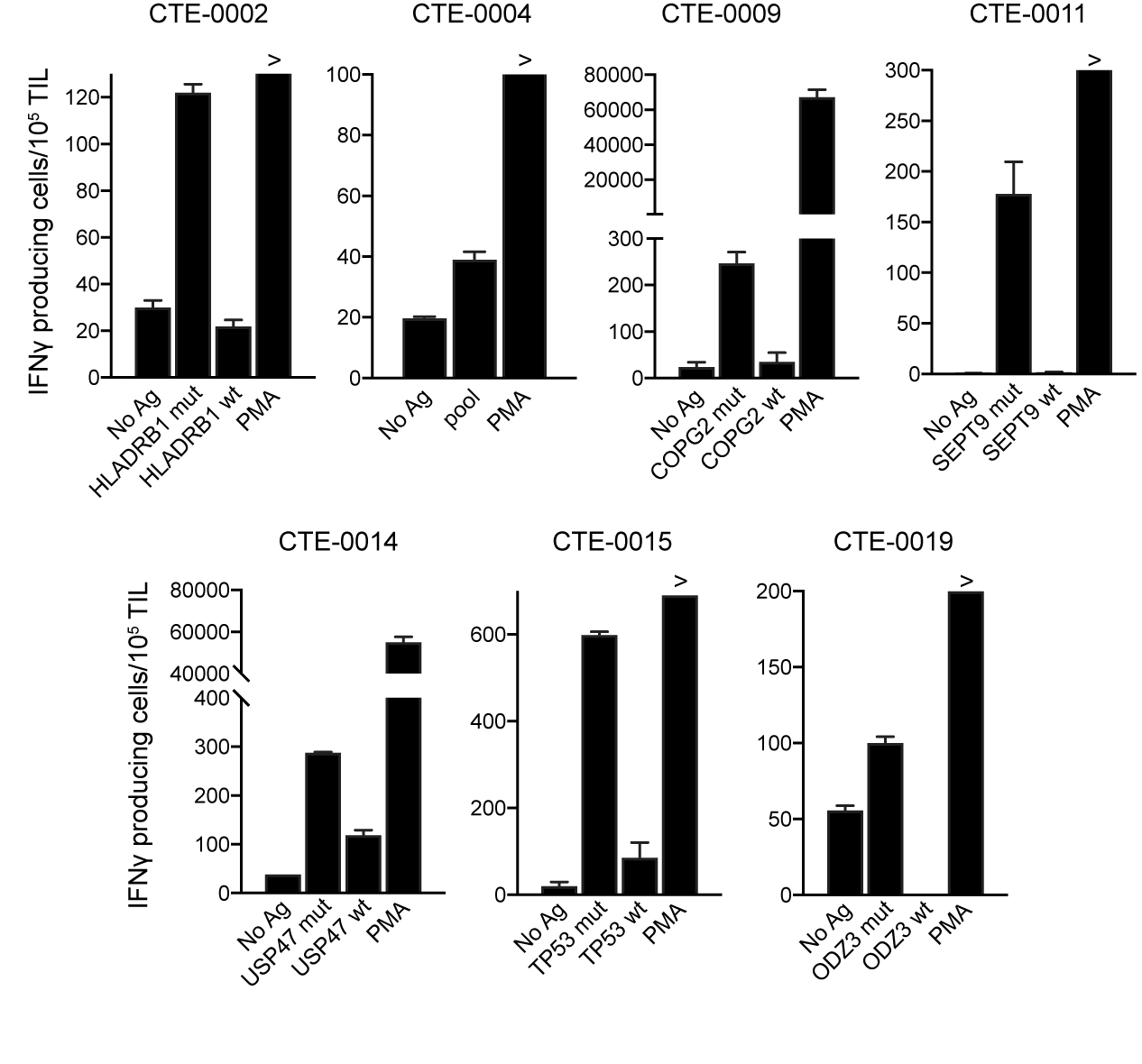
**

**Supplementary Figure 3. Validations of TIL CD8^+^ T-cell responses against neo-epitopes and native (wild-type) peptides**

Shown are the numbers of IFNγ-producing TILs determined by ELISpot (average of triplicates + SD). Neo-epitopes are described in Supplementary Table 2. All examples of neo-epitope validation shown were obtained with the primed TIL expansion protocol with the exception of CTE-0002 (which was only tested with the conventional TIL expansion protocol).

**Supplementary Figure 4**

**
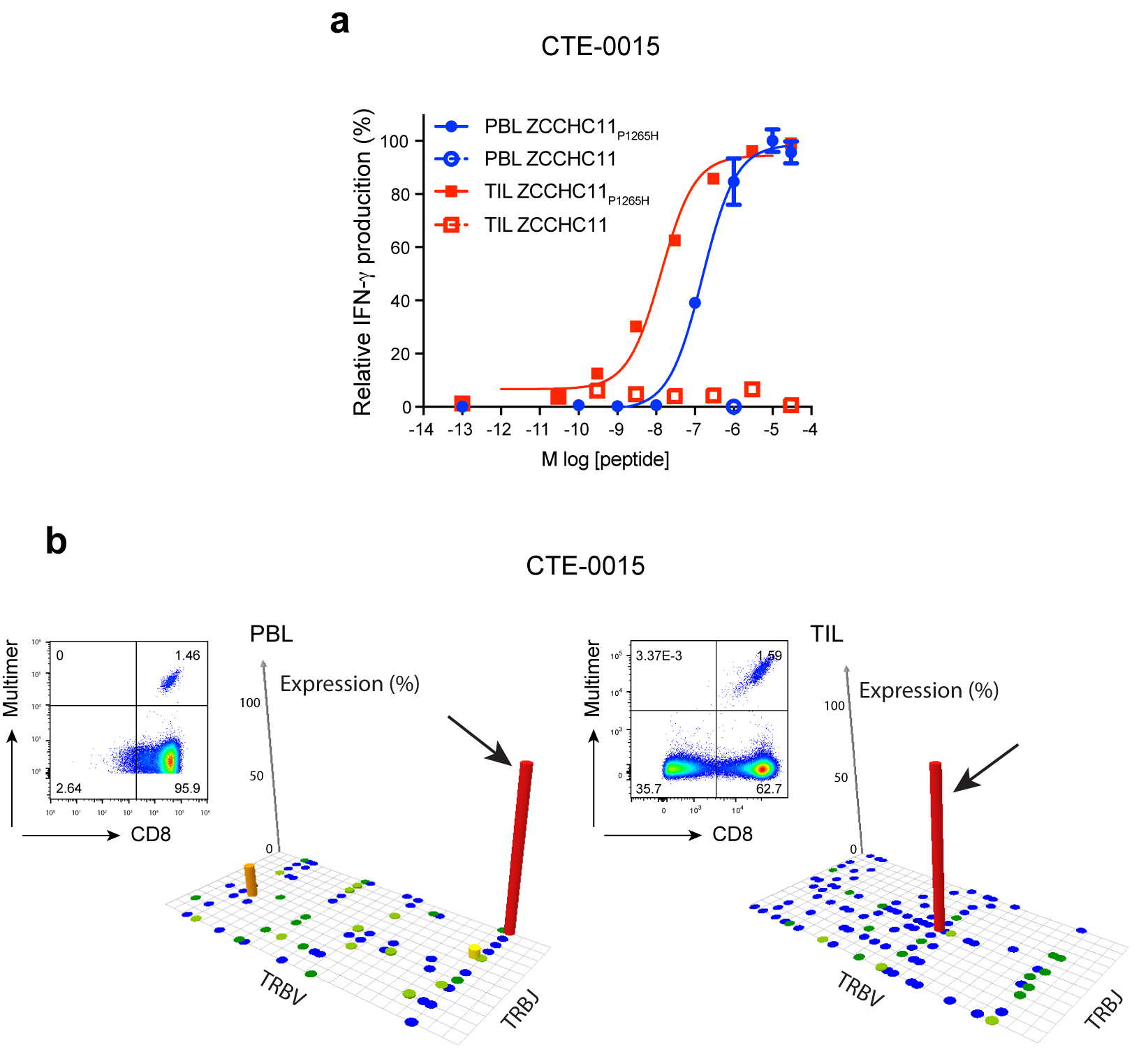
**

**Supplementary Figure 4. Higher functional avidity of TILs relative to PBLs recognizing the same neo-epitope in patient CTE-0015**

**a**) Representative example of functional avidity of *ZCCHC11_P1265H_*-specific clones obtained from TILs and PBLs, as assessed by limiting peptide dilution in IFNγ ELISpot. **b**) Analysis of the TCRβ repertoire of *ZCCHC11_P1265H_*-specific CD8^+^ T cells purified by FACS sorting with multimers from PBLs (left) and TILs (right) in patient CTE-0015.

**Supplementary Figure 5**

**
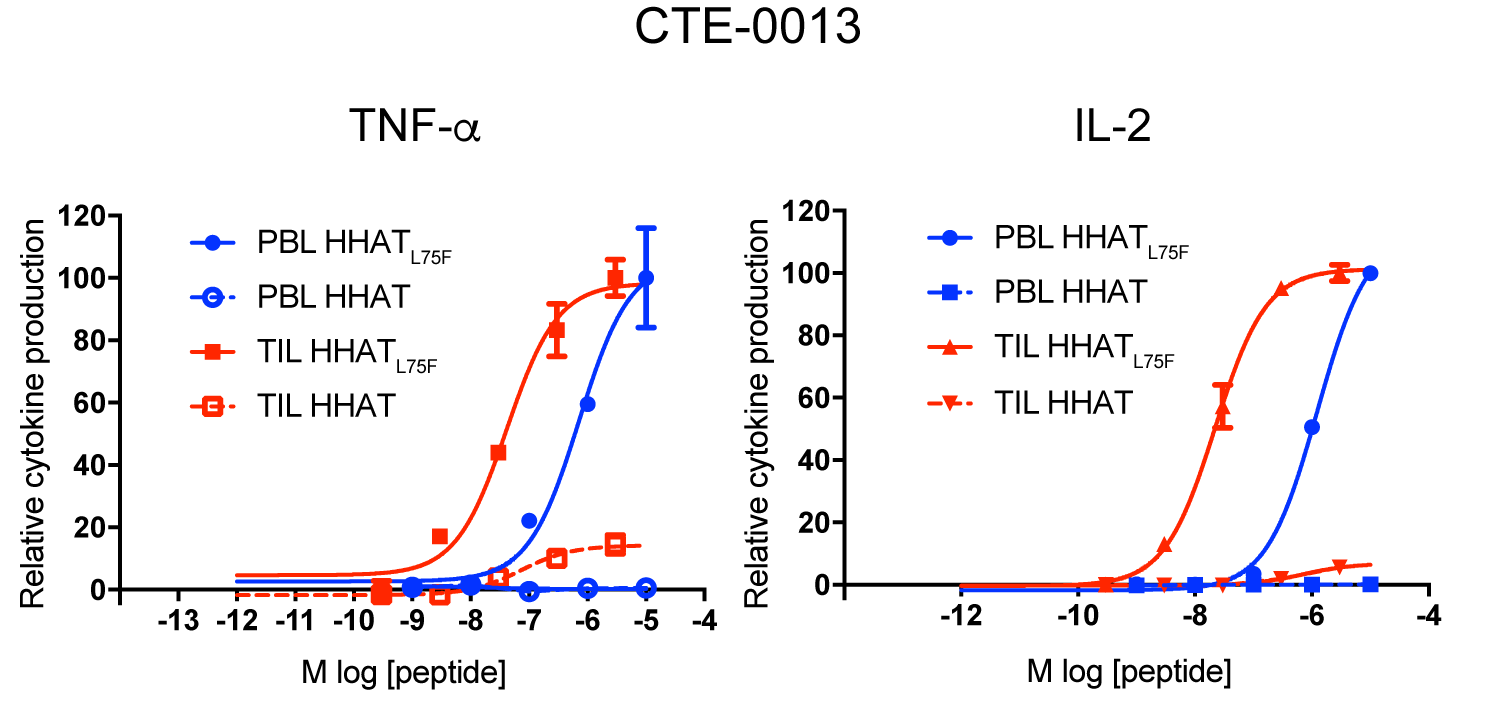
**

**Supplementary Figure 5. Antigen sensitivity of TILs relative to PBLs recognizing the same neo-epitope in patient CTE-0013**

Representative examples of the higher functional avidity of *HHAT_L75F_*-specific T cells isolated from tumors (TILs) compared to blood (PBLs). Shown are the relative concentrations of TNFα and IL-2 as determined by multiplex MSD.

**Supplementary Figure 6**

**
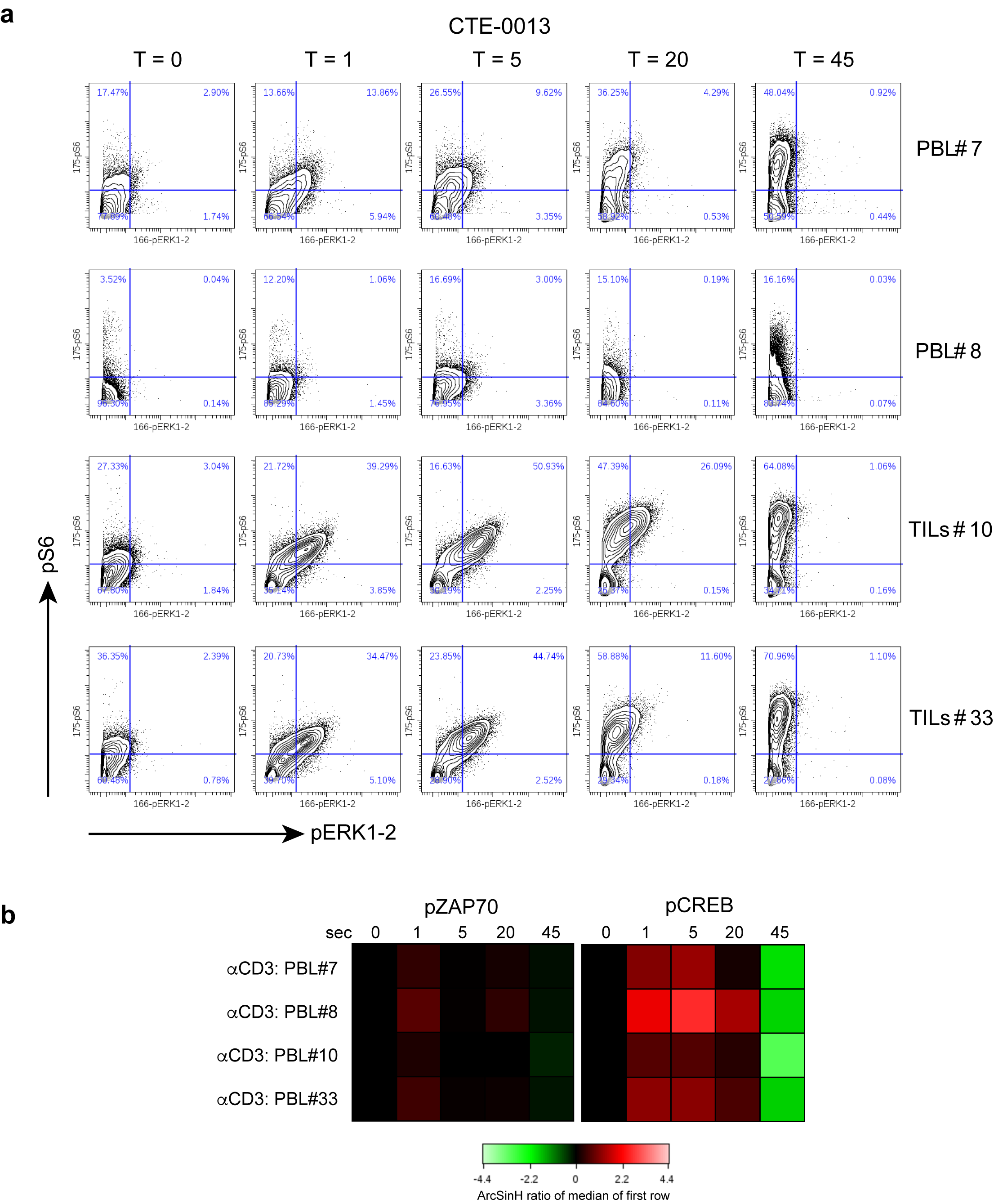
**

**Supplementary Figure 6. Analyses of TCR signaling pathways of neo-epitope specific PBLs and TILs in patient CTE-0013**

**a**) Raw data showing the stronger and faster activation of ERK1/2 and S6 in *HHAT_L75F_*-specific TIL relative to PBL clones. **b**) Lack of difference in early signaling events, assessed by ZAP70 and CREB phosphorylation, between *HHAT_L75F_*-specific T cells isolated from TILs and PBLs.**Supplementary Figure 7**

**
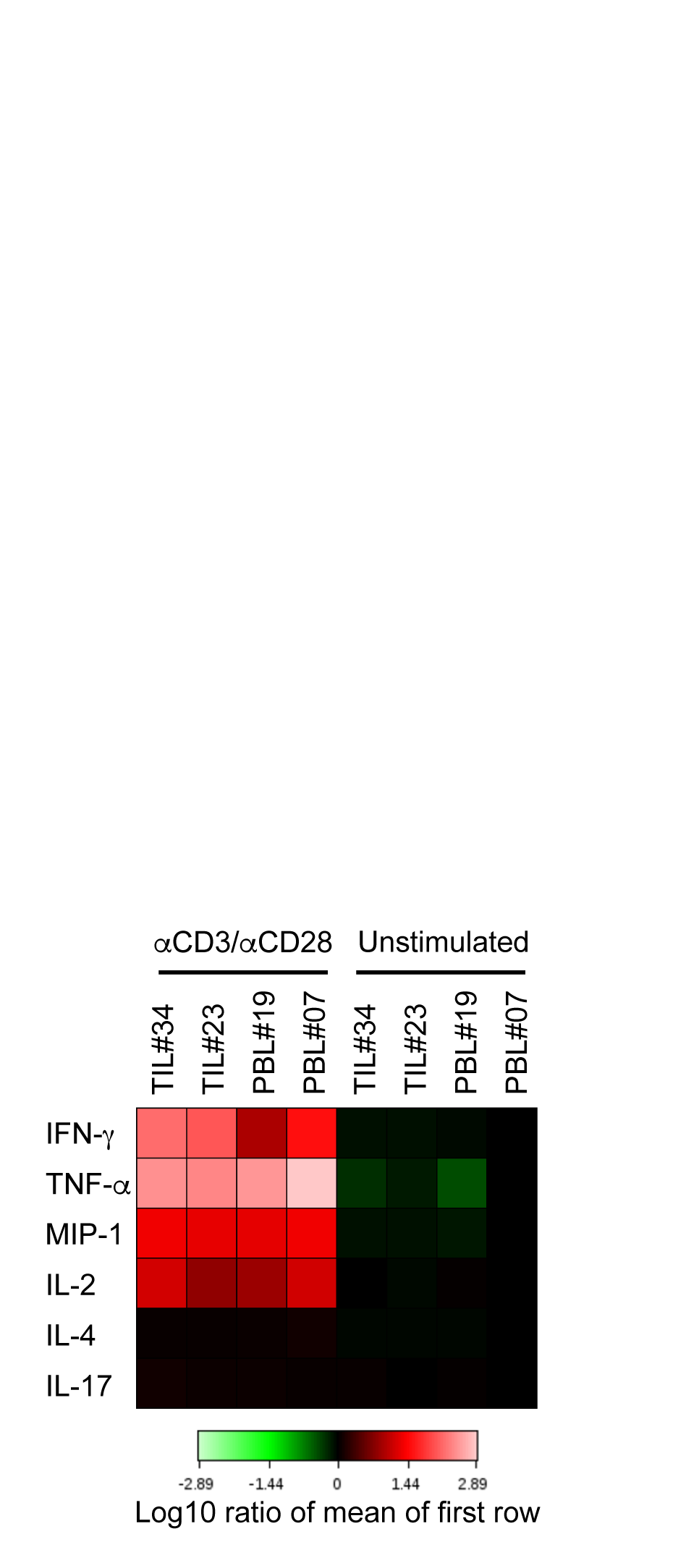
**

**Supplementary Figure 7. Relative production of cytokines by neo-epitope specific PBLs and TILs in patient CTE-0013 following CD3/CD28 stimulation**

Mass cytometry analysis shows the relative production of cytokines by *HHAT_L75F_*-specific PBLs and TILs. Unstimulated controls are also shown for each population.

**Supplementary Figure 8**

**
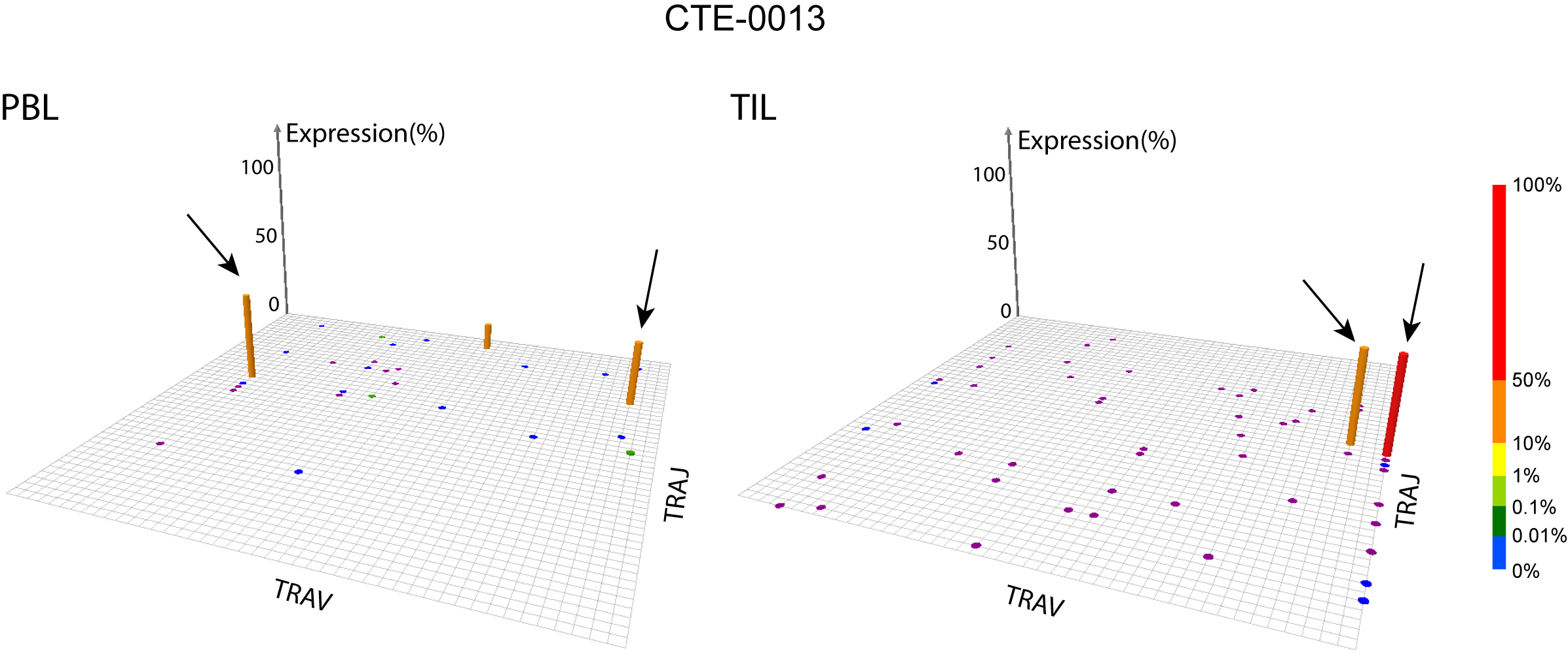
**

**Supplementary Figure 8.** **Analysis of the repertoire of the T-cell receptor α (TCRα) V-J segment recombination of *HHAT_L75F_*-specific CD8^+^ T cells isolated from PBLs and TILs** *HHAT_L75F_*-specific CD8^+^ T cells were purified by FACS sorting using multimers (Fig. 4f). V and J segments are represented according to chromosomal location on the *x* and *y*-axis, respectively; the frequency of each recombination is shown on the *z*-axis (height) and highlighted by colors.

**Supplementary Figure 9**

**
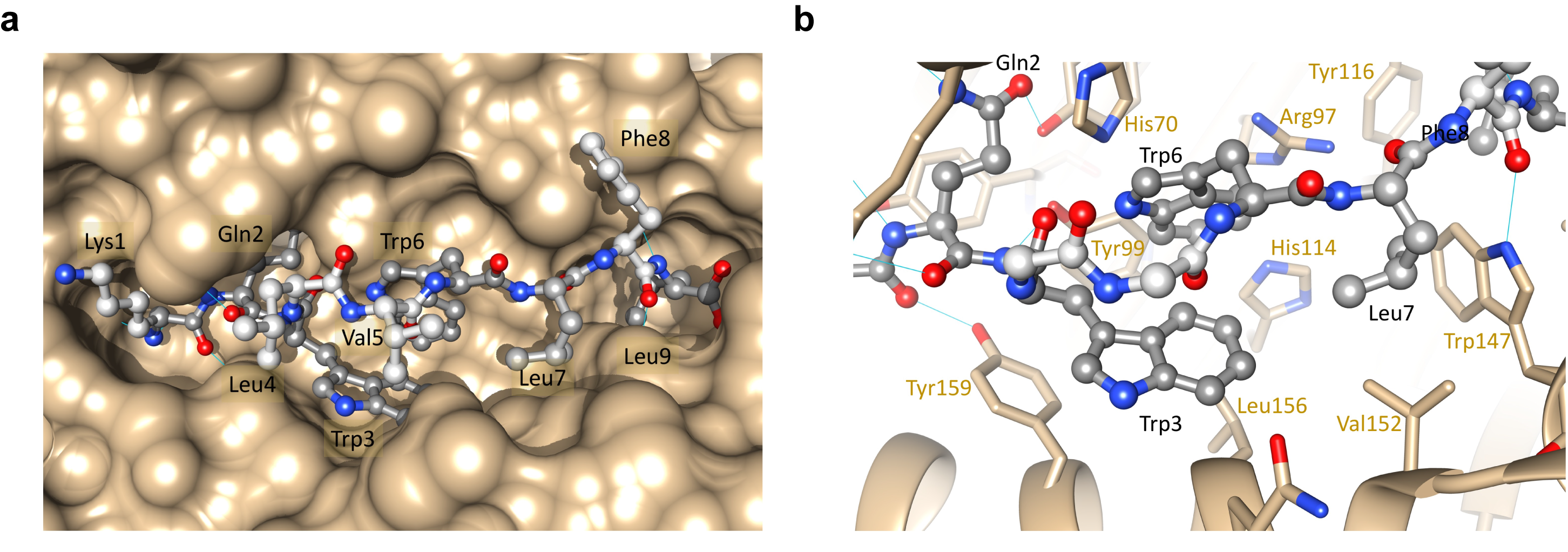
**

**Supplementary Figure 9. Calculated binding mode of peptide KQWLVWLFL (*HHAT_L75F_*) on HLA-A*0206.** The peptide is shown in ball and stick, colored according to the atom types. Carbon atoms are colored in dark grey for buried residues, and in light grey for residues exposed to the TCR (Lys1, Leu4, Val5 and Phe8). The binding mode was taken from the complex with TCR *HHAT*-TIL (hTRAV38-2 + hTRAJ33 / hTRBV12-3 + hTRBJ02-3). The TCR was removed for the clarity of the figure (the figure shows the pMHC surface exposed to the TCR). Trp3 and Trp6 are predicted to be buried in the same – essentially aromatic – pocket. Anchor residues Gln2 and Leu9 are deeply buried in the MHC surface. b) Same binding mode, centered on the peptide Trp3 and Trp6 residues. MHC residues are shown in thick lines, colored according to the atom types, with carbon colored in brown. The TCR, as well as the side chains of peptide residues Leu4 and Val5 were removed for the clarity of the figure. Peptide and MHC residues are labeled in black and brown, respectively. The predicted molecular interactions between the *HHAT_L75F_* peptide and HLA-A*0206 were similar for all four TCR-pMHC complexes analyzed. They included the well-known canonical hydrogen bonds between the N-terminus ammonium function and the side chains of MHC Tyr7, Tyr159 and Tyr171 on one side, and between the C-terminus carboxylate function and the side chains of MHC Tyr143, Lys146 and hb-Tyr84 on the other side. The side chain of the N-terminal anchor residue Gln2 was buried and made hydrogen bonds with the side chains of MHC Tyr9 and Glu63, and with the backbone carbonyl of Glu63. The side chain of the C-terminal anchor residue Leu9 was also buried in MHC and occupied a non-polar pocket constituted by the side chains of MHC Leu81, Tyr116, Thr143 and Trp147. Interestingly, the side chains of both Trp3 and Trp6 were predicted to be buried in the same non-polar and essentially aromatic pocket, constituted of MHC residues His70, Tyr99, His114, Leu156, and Tyr159, with which they can make several π-π interactions. Trp6 also made a cation-π interaction with MHC Arg97. In addition, Trp3 and Trp6 made π-π interactions together. Peptide Leu7 was predicted to be partially buried in a non-polar pocket formed by MHC Trp147 and Val152.

**Supplementary Figure 10**

**
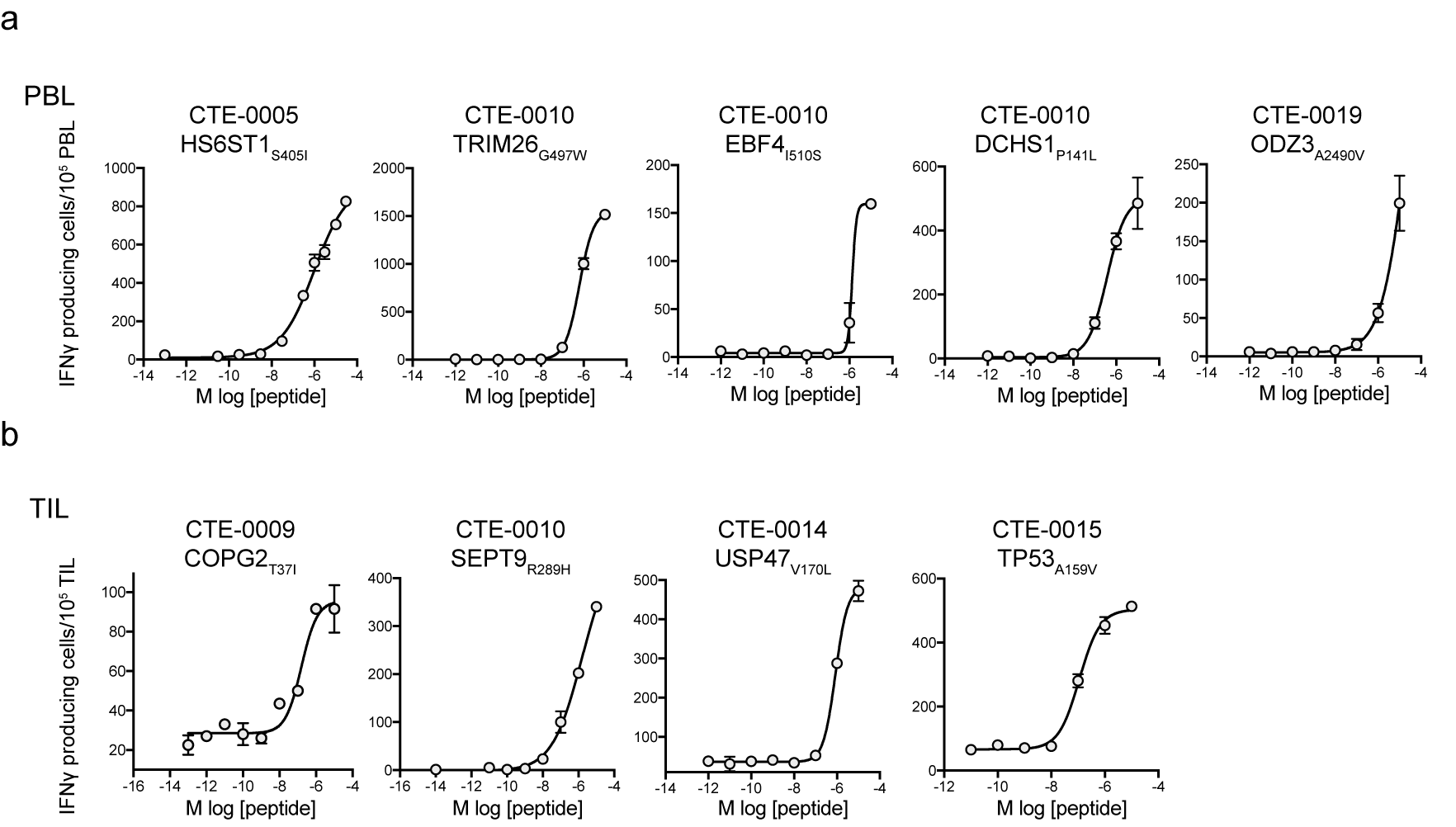
**

**Supplementary Figure 10. Antigen sensitivity of neo-epitope specific T cells from PBLs and TILs**

Raw data of representative experiments (n ≥ 2) showing the functional avidity of all neo-epitope specific CD8^+^ T-cell responses shown in Fig. 5a.

**__**

**Supplementary Table 1a.** Description of immunotherapy-naïve patients with recurrent EOC. Patient identification number, age, tumor histologic type (ovarian: epithelial ovarian cancer; peritoneal: primary peritoneal serous carcinoma), FIGO stage, number of type of prior chemotherapy lines (chemos), undergoing treatments and inflammatory condition, tumor histology, number of prior recurrences, tumor grade and BRCA1/2 and HRD status.

**Supplementary Table 1a**

**Supplementary Table 1b**

**Supplementary Table 1b**

Total number of non-synonymous somatic mutations, HLA class-I haplotypes and number of predicted neo-epitopes.

**Supplementary Table 2**

**Supplementary Table 2**

Description of the 15 validated HLA class-I neo-epitopes. Patient identification number; mutated gene; specific mutation identified; HLA class-I restriction; peptide sequence and predicted affinity of neo-epitope (binding score predicted by NetMHC algorithm).

**Supplementary Table 3**

Pathways differentially expressed in patients with neo-epitope specific PBLs.

**Supplementary Table 4**

**Supplementary Table 4**

Protein Data Bank entries used as templates to model the TCRα, TCRβ, MHC, β-microglobulin and the peptide epitope.

**Supplementary Table 5**

**Supplementary Table 5**

Molecular interactions between the peptide epitope (KQWLVWLFL) and HLA-A*0206, as predicted by homology modeling. bb: backbone, sc: side chain; hb: hydrogen bond, io: ionic interaction; np: non-polar interaction, π: π-π interaction, cπ: cation-π interaction. Unless indicated, interactions are taking place between side chains.

**Supplementary Table 6.**

Molecular interactions between PBL and TIL TCRs and pMHC, as predicted by homology modeling. bb: backbone, sc: side chain; hb: hydrogen bond, io: ionic interaction; np: non-polar interaction; π: π - π interaction.

**Supplementary Methods**

CyTOF analysis: Clones, sources and concentrations of the antibodies used for extracellular and intracellular stainings.
